# A follow-up study on Guillain-Barre syndrome and validation of Brighton criteria

**Published:** 2019-04-04

**Authors:** Reza Boostani, Farveh Ramezanzadeh, Morteza Saeidi, Mina Khodabandeh

**Affiliations:** 1 Department of Neurology, School of Medicine, Mashhad University of Medical Sciences, Mashhad, Iran; 2 Inflammation and Inflammatory Disease Research Center, Mashhad University of Medical Sciences, Mashhad, Iran

**Keywords:** Guillain-Barre Syndrome, Prognosis, Diagnosis, Nerve Conduction Study, Iran

## Abstract

**Background:** Guillain-Barre syndrome (GBS) is the major cause of acute flaccid paralysis (AFP). Comprehensive classification and predictive measures need to be created for GBS. This study was conducted to evaluate GBS patients’ prognosis and Brighton criteria validity in Iranian population.

**Methods:** This retrospective cohort study was conducted using medical records of patients with GBS admitted to Ghaem Hospital, Mashhad, Iran. After collecting data from cerebrospinal fluid (CSF) analysis, nerve conduction studies, and clinical examinations, Brighton criteria and GBS disability scores were calculated. Patients ultimately received follow-up telephone calls after 15 to 45 months of admission, checking on one’s clinical status and the ability to walk independently. Data were analyzed using SPSS software.

**Results:** Patients were mostly men (78.0%) with the mean age of 48.58 years. GBS onset was reported more frequently in spring. According to Brighton criteria, 41.4%, 51.6%, and 7.0% of the patients were classified as levels 1, 2, and 4, respectively. For GBS disability score, 54.7%, 16.4%, 9.4%, and 6.2% of the patients had grades of 4, 3, 2, and 1, respectively. 37 patients (39.4%) restored the ability to walk within the first month, while 3 patients (3.2%) were unable to walk by the end of the second year. Significant relationship was observed between the ability of walking independently and GBS disability score (P < 0.001).

**Conclusion:** In the Iranian GBS population, less than half of the patients met level 1 of Brighton criteria and more than half of them reached the GBS disability score of 4, and walking ability was correlated to GBS disability score.

## Introduction

Guillain-Barre syndrome (GBS) is a medical emergency characterized by an acute peripheral neuropathy including a rapidly-developing motor and sensory weakness.^1^

It has been estimated that the overall incidence of GBS ranges from 1.1 to 1.8 per 100000 persons/year, increasing in the population of 50 years of age and more to 3.3 per 100000 persons/year.^2^ This life-threatening disorder can even cause mortality and morbidity despite medical support. The patients can die regardless of the disease stage, even after being discharged from intensive care unit (ICU). Having a high burden of disease reveals the necessity for developing accurate diagnostic measures and identifying factors that affect the prognosis of GBS.^3^ Diagnosis of GBS has been a challenging issue, as early diagnosis can improve disease outcomes. Various sets of diagnostic criteria have been established since the identification of first case of the disease in 1916.^4^ Suggested in 1976, the first set of diagnostic criteria was reliable for detecting suspicious cases with muscle impairment after vaccination in national influenza immunization program.^5^ In 1978, Hughes, et al. proposed a disability scale which has been used widely till now for GBS diagnosis.^6^ Twelve years later, the criteria was revised to be applicable for research purposes.^7^ Recently, the Brighton Collaboration has proposed a more valid set of criteria for identifying suspected cases.^8^ The relationship between GBS outcomes and different GBS criteria or clinical findings has still remained unclear. In this study, the correlation between the two-year outcome of patients with GBS and their disease manifestations has been assessed considering disease severity and diagnostic certainty scores.

## Materials and Methods

The present retrospective cohort study was conducted in neurology ward of Ghaem Hospital in Mashhad, Iran. This study was approved as a resident thesis by the Ethics Committee of Mashhad University of Medical Sciences, Mashhad (code: 960279). We used the medical records of patients with GBS admitted to the hospital from September 2016 to September 2017, all of which fulfilled the criteria of National Institute of Neurological Disorders and Stroke (NINDS). We also applied the medical records of patients with GBS who were enrolled in a previous study (March 2015-August 2016),^9^ performed by the same research group. All of the patients were older than 14 years of age for whom GBS was diagnosed by a neurologist, after performing neurologic examinations and further required laboratory tests. 2 patients with Miller Fisher syndrome (MFS), 7 patients with chronic inflammatory demyelinating polyneuropathy (CIDP), and 4 other patients with brucellosis, vasculitis, meningeal infiltration, and paraneoplastic neuropathy were excluded from the study. After obtaining informed consent, every patient underwent a complete physical examination performed by a neurology resident and demographic data including age, gender, history of any previous medical illness, and the results of their laboratory workups were documented. Available cerebrospinal fluid (CSF) analysis results were also documented for cellular count and protein and glucose levels. Time from symptoms onset and initiation of the severe phase was recorded. The nerve conduction study (NCS) was performed for all patients after a mean period of 5.3 days of symptoms onset. Categorization at the time of the hospitalization was performed according to Brighton diagnostic criteria and GBS disability scoring. Telephone-call fallow-up for checking on the clinical status and the ability to walk was performed at least 15 months after admission. Patients diagnosed with other clinical illnesses during the follow-up period were also excluded from this study. Finally, data were analyzed using SPSS software (version 16, SPSS Inc., Chicago, IL, USA).

## Results

The mean and standard deviation (SD) of age and admission duration of patients with GBS were 48.58 ± 21.09 years and 18.45 ± 24.10 days, respectively. Most of the patients were men (78 vs. 50). Mean time from disease onset to lumbar puncture was 9.98 ± 6.47 days. The clinical findings are summarized in table 1 and patients^,^ classification according to Brighton criteria is demonstrated in table 2.

**Figure 1:**
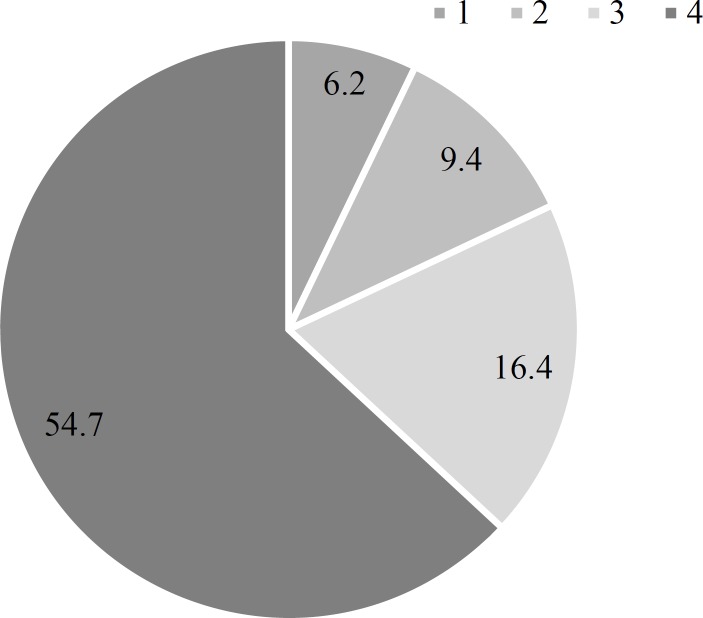
Percentage of different Guillain-Barre syndrome (GBS) disability scores at the time of admission

Among the clinical findings, CSF protein levels, pain, and cranial nerve involvement were not considerably different between the genders (P = 0.597, P = 0.275, and P = 0.189, respectively). Among the study population, a concurrent lymphoma, a history of leukemia, and a history of previous GBS were detected and one patient developed leukemia a year after GBS diagnosis. The GBS disability score at the time of admission is illustrated in figure 1. 

The highest onset rate of GBS was observed in spring, followed by winter, summer, and fall. During admission, 12.5% had autonomic dysfunction, mostly bladder dysfunction (4.0%).

**Table 1:** Clinical findings of patients with Guillain-Barre syndrome (GBS) at the time of the diagnosis

**Clinical findings**		**Percentage**
Pain		15.6
Cranial nerve involvement	Facial	23.2
	Bulbar	14.7
	Ophthalmic	4.5
Sensory involvement	Sensory level	3.9
	Hypoesthesia	2.3
	Paresthesia	8.6
Autonomic dysfunction	Blood pressure rising	3.1
	bladder dysfunction	4.0
	Tachycardia	3.9
	Bradycardia	0.8
	Hypotension	0.8
History of previous infection	No infection	48.4
	Upper respiratory tract infection	34.4
	Gastroenteritis	14.1
	Vaccination	1.6
	Other infection	1.6
NCS study	AIDP	70.3
	AMAN	5.5
	AMSAN	7.8
	Normal	7.0
	Other	9.4

NCS: Nerve conduction study; AIDP: Acute inflammatory demyelinating polyneuropathy; AMAN: Acute motor axonal neuropathy; AMSAN: Acute motor sensory axonal neuropathy

The most common NCS findings were acute inflammatory demyelinating polyneuropathy (AIDP) (70.0%), acute motor sensory axonal neuropathy (AMSAN) (7.8%), and acute motor axonal neuropathy (AMAN) (5.5%). 26 (20.3%) patients required ICU admission and mechanical ventilation was provided for 22 patients (17.2%). Majority of the patients had high protein levels in CSF. There was not any significant difference in CSF protein levels between the genders (P = 0.597). Intravenous immunoglobulin (IVIG) (48.4%) and plasmapheresis (32.0%) were the most frequently used therapeutic modalities, both of which were applied as combination therapy for 14.0% of the patients. One patient received methylprednisolone pulse therapy and 4.7% did not receive any specific treatment. 94 patients (73.4%) responded to follow-up telephone calls after 2 years of admission from which, the ability to walk in the first place and after a month of disease onset was reported in 6 (6.4%) and 31 (33.0%) patients, respectively.

**Table 2:** Brighton criteria and its association with other clinical findings

**Brighton ** **score**	**Percentage**	**Gender (%)**		**Prognosis (%)**		**NCS findings (%)**				
		**Men**	**Women**	**Good**	**Poor**	**AIDP**	**AMAN**	**AMSAN**	**Normal**	**Other**
1	41.4	50.1	49.1	87.2	12.8	79.2	3.8	9.4	5.7	1.9
2	51.6	65.5	34.8	74.5	25.5	66.7	7.6	6.1	6.1	13.6
4	7.0	88.9	11.1	100	-	44.4	-	11.1	22.2	22.2

NCS: Nerve conduction study; AIDP: Acute inflammatory demyelinating polyneuropathy; AMAN: Acute motor axonal neuropathy; AMSAN: Acute motor sensory axonal neuropathy

**Figure 2:**
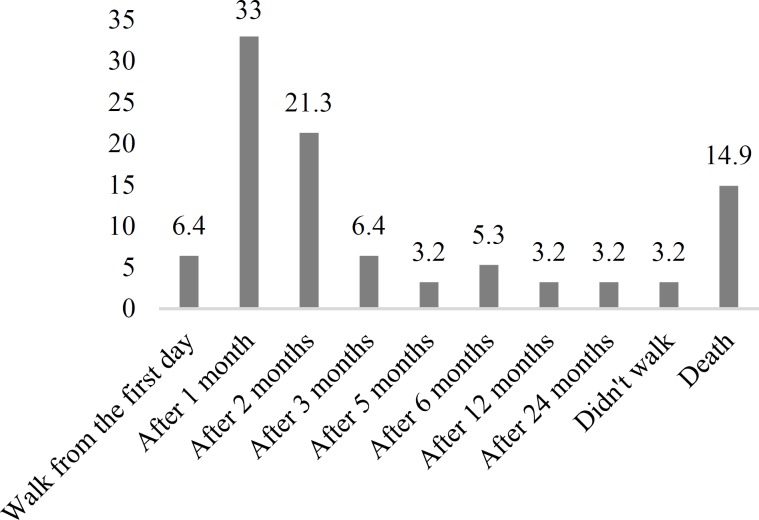
Walking ability during the follow-up period

14 patients (14.9%) had died and 3 patients (3.2%) had not restored walking ability after 2 years (Figure 2).

Those who restored walking ability any time after disease onset were categorized as the “good prognosis” group, and those who died or were unable to walk were categorized as the “poor prognosis” group. Mann-Whitney test showed a significant relationship between prognosis categories and GBS disability scores (P < 0.001). The mean time from disease onset to the lumbar puncture in those who had high protein levels and normal protein levels was 10.3 and 9 days, respectively (P = 0.435).

## Discussion

GBS can be life-threatening if left untreated. Alter has reported that approximately 75% of patients with GBS would completely recover.^10^ Our study in the referral center showed that most of the patients with GBS had GBS disability scale of 4, representing their poor outcome and ability to walk independently.

GBS characteristics vary across different parts of the world and some of the findings seem to be more common in specific populations. Majority of the cases in our study were men (60.9%). This gender dominancy has been also reported in previous studies from Isfahan^11^ and East Azerbaijan,^12^ Iran, while 2 other studies from Shiraz, Iran, have reported the opposite.^13^ The gender did not have significant effects on clinical findings of our study including cranial nerve involvement, pain, and CSF protein, while some studies showed a relationship between gender and manifestations like cranial nerve involvement.^14^ Seasonal prevalence of GBS and the most common proceeding illness in our research were similar to the other Iranian studies, demonstrating the dominancy of spring and winter and the importance of the proceeding upper respiratory tract infection.^15^ Infections which can provoke immune response in human body, especially against nervous system, are considered as possible triggers for GBS development. Immunization can also induce GBS which was reported in 1.6% of our patients. The AMSAN sub-type of GBS has been reported to be related to infections, especially those affecting respiratory tract.^11^ Moreover, 7.0% of our patients had normal NCS findings which could be a result of early diagnostic workup. In our study, AIDP was the most common sub-type detected by NCS. Arami, et al. from northwest of Iran have also reported that demyelination is the most common electrodiagnostic (EDX) characteristic found in GBS.^12^ These findings are not in line with Ansari, et al. study which has reported AIDP as the third common sub-type. This could be explained considering the large portion of unavailable reports in their study.^11^ Grapperon, et al.^11^ and Yadegari, et al.^16^ have recently provided a useful electrophysiological classification of GBS according to different sets of diagnostic criteria. Among their 69 patients with classic GBS, the Hadden, et al.’s criteria^17^ had more demyelinating cases followed by equivocal ones, while the Rajabally, et al.’s criteria^18^ had more demyelinating cases followed by axonal ones.

The other important issue is the outcome of GBS. Fokke, et al. reported that after 6 months, 82.0% of their study population could walk independently, while 3.0% of them had died.^8^ According to our study results, 62.9% of our patients were able to walk after 6 months and 14.9% of them had died. Results from our follow-up for maximum of 45 months considering the ability of walking were not similar to those from 6 months fallow-up in Fokke, et al. study.^8^ This difference can be a result of their different treatment regimens which were mostly IVIG (49.0%) followed by IVIG and methylprednisolone, while the second most common treatment used for our patients was plasmapheresis and only 14.0% of our total population received a combination of IVIG and plasmapheresis. Arami, et al. also applied a different regimen for the Iranian population with GBS in their study which was plasma exchange as the most common treatment followed by IVIG.^12^ A recent review about the efficacy and superiority of plasmapheresis to IVIG in GBS did not reveal significant differences and both of these treatments could hasten the recovery of such patients.^19^ Moreover, it seems that corticosteroids administration alone may not promote the recovery of patients with GBS or affect their long-term outcomes.^20^ Respiratory support can reduce the adverse outcomes if provided in time. Utilizing ventilation support and cranial nerve involvements was reported in 10.0% and 36.0% of the cases in Fokke, et al.,^8^ and 17.2% and 32.8% of the patients in our study, respectively. It has also been reported that mechanically ventilated patients had higher GBS disability scores.^12^ Protein concentrations of CSF raised with the time from the GBS onset to the lumbar puncture, as demonstrated in Fokke, et al.^8^ study too, where 64.0% of their population had elevated CSF protein level, increased to 88.0% within the first 3 weeks of the onset. 

As mentioned before, our patients were mostly categorized as level 2 and 1 according to Brighton score, emphasizing the fact that in contrast to a similar study,^21^ we had less unavailable data and more completed investigations in our center. 41.4% of our patients have been categorized in level 1 which was more suggestive of GBS with the most completed diagnostic workups. Fokke, et al. demonstrated that 61.0% of their patients were with complete records and 41.0% of all their patients had level 1 of Brighton criteria.^8^ Islam, et al.^22^ and Grapperon, et al.^14^ reported that 58.0% and 49.0% of their patients had level 1 criteria, respectively. Mateen, et al. stated that 62.0% of their patients had level 1 of Brighton criteria, and that the sensitivity of level 2 and 1 were 83.0% and 62.0%, respectively.^20^ Such findings highlight the fact that Brighton criteria provides variable results in different populations with different diagnostic settings. Alongside with Brighton criteria, GBS disability score in our population was similar to the most of the other studies. The GBS disability scale of Fokke, et al. population was the same as ours and their most common score was also 4 followed by 3.^8^ Arami, et al. reported that most of their patients had GBS disability score higher than 3 and after 6 months, 19.7% of their patients still had high score. They could not relate the high GBS disability score to demyelinative or axonal damage or old age (> 50 years), but they found significant relationship between axonal damage and the history of diarrhea, with the worst outcomes after 6 months.^12^

## Conclusion

According to our results, more than half of our patients with GBS were categorized as Brighton level 2 and more than half of our patients had GBS disability score of 4. Even though applying Brighton criteria has been suggested for vaccine safety studies, it seems that decision making based on this criteria requires further studies in clinical settings.

## Notes:

**How to cite this article:** Boostani R, Ramezanzadeh F, Saeidi M, Khodabandeh M. A follow-up study on Guillain-Barre syndrome and validation of Brighton criteria. Iran J Neurol 2019; 18(2): 64-9.
